# A Case of Cholera, with Remarks

**Published:** 1849-08

**Authors:** R. L. Scruggs

**Affiliations:** Germantown, Tenn.


Art. VI.—*A Case of Cholera, with Remarks.* By R. L. Scruggs, M. D., o Germantown, Tenn.

At 8 o'clock on the morning of the 20th of June, I was called in consultation to a man who had arrived in our village and been taken sick about an hour before. The patient, æt. 35, was a cattle driver from North Mississippi, who, during a few days' stay in Memphis, lodged in a house near the Bayou, where cholera had prevailed from its commencement with greater violence and fatality than in any other portion of the city. He had labored, as I afterwards learned, under diarrhea for several days before. When I saw him he had had several serous dejections in quick succession, accompanied by pretty violent vomiting of watery matters, and cramps. His face wore an exceedingly anxious expression (occasioned probably partly by fear); his extremities were cold; the surface of his whole body bathed in sweat and suffering from great prostration of the vital energies. Pulse 128 and very feeble. Except a small quantity of blue pill and Dover's powder he had taken nothing previous to my arrival. The following prescription was agreed upon.

℞ Hydrarg. submur. grs. x.
Capsic.
Plumb acet. aa. grs. v.
M. To be taken immediately.

In addition to this, sinapisms were applied along the whole course of the spine, over the abdomen and lower part of the chest, and his extremities were well rubbed with equal parts of pepper and mustard. After the lapse of half an hour I gave him 40 drops of camphor dissolved in chloroform, prepared according to Dr. T. S. Bell's formula, which almost immediately caused his countenance to brighten up, and produced a most agreeable sensation of warmth about the stomach. This was repeated once or twice with the same happy results, and, in addition to which, it reduced the frequency and increased the force of the pulse.

We now left the patient, directing the calomel, sugar of lead and capsicum to be repeated every two hours, but in smaller quantity than at first; the perspiration to be wiped off constantly with a coarse cloth; the sinapisms to be reapplied occasionally, the extremities to be rubbed with the dry mustard and pepper; and the chloroform and camphor to be administered as before, provided the vomiting should at any time recur, or there should be anything like collapse or sinking, or cramps.

At 8 o'clock in the evening, when I saw him again, although his whole body, particularly his extremities, felt cold and shriveled, yet there was an improvement, (pulse 112 and stronger); and when I was informed that the patient had discharged about a pint of dark colored consistent bile from his bowels, I felt perfectly satisfied with his condition.* During the night he had two other bilious passages, and when I saw him early the next morning reaction was perfect. The patient himself expressed astonishment at his strength compared with the great prostration the day before; said he felt quite well, and asked for some food, which was allowed him in the shape of chicken broth. The after treatment consisted in a small dose of hydrarg. cum. cret., assisted by a mild purgative. In three days afterwards the patient started home, and I have not since heard from him.

It will be observed that no brandy or opium was used in this case, except the small quantity of Dover's powder at the commencement. I think that the chloroform and camphor, or chloroform alone will be found to be an admirable substitute for these articles, producing no narcot-

---

* Durrng the epidemic of 1832 and 33, I saw Dr. Rose treat a number of cases of cholera at Memphis, and with more success than any physician there; and although at that time, I had not even commenced the study of medicine, I distinctly recollect that he mainly relied upon small doses of calomel repeated at short intervals; and when he succeeded in bringing away "*black* bile," which he rarely failed to do, he pronounced his patient safe, and in this he was never mistaken. At the same time I saw other patients die with more than two thousand grains of calomel in them, administered in doses of from 60 to 80 grains, without even having a single bilious discharge.

ism, nor any after depression, such as invariably follows the free use of alcoholic stimuli. I would not have it inferred from this, however, that I think that these articles, particularly opium, could in no case be advantageously used; but I do believe that a vast deal more mischief has been done with them, in this epidemic, than good. Indeed I feel perfectly confident that I have seen recently several persons in Memphis, in a dying condition from the effects of brandy and opium, and have heard of many other similar cases from some of the most respectable physicians of that place. For the extensive mischief done during the present epidemic with these two articles, I think we are mainly indebted to the injudicious essays of Doctor Hawthorn of Liverpool, Eng., republished by the public press in this country.

Cholera has prevailed in Memphis about fifteen miles distant from this place, from early in January to the present time. Its progress and intensity have more than once sensibly fluctuated since its advent. Several times it was thought to have disappeared when it would again recur with apparently increased malignity. No case that I am aware of has as yet originated in the country, though several persons after leaving the city, have been attacked, some of whom have died.

Diarrhea, dysentery and severe cholera morbus have all been more prevalent here this than for many previous years; but, so far as I am informed, little fatality has attended either disease. Notwithstanding which, it will not be considered unphilosophic, I presume, to conclude that there exists some relationship between the remote and proximate causes of these diseases, and genuine Asiatic cholera, especially when we observe that they prevail epidemically, previous to the appearance of cholera. Indeed I have been disposed to extend this idea still further back, and include influenza and its accompanying cerebro-spinal affections, in the same category: but my ideas upon this obscure subject are not sufficiently matured for me to venture a positive opinion upon it. That they have been precursory of the disease in many, if not all of the places visited by cholera, is a fact well calculated to cause a suspicion, at least, of a common origin; and ought certainly to excite inquiry upon the subject. Even should this inquiry result in a failure to detect the true etiology of cholera, yet if we succeed in distinctly recognizing the diseases which precede and follow it (if indeed there really be any uniformity in this), we will have gained that which will undoubtedly materially assist us in our precautionary efforts, and sanitary means to be used before, during and after the prevalence of this destructive malady.

That the disease sometimes commences in so mild a form as to cause doubts in the minds of even medical men, as to the diagnosis; gradually sometimes, but often rapidly increasing in virulence until it attains its climax—then gradually becoming more and more mild until it entirely disappears—are facts connected with its history, which go strongly to favor the idea, that the diseases which resemble, precede and follow cholera, are produced by the same morbific agency in a *less concentrated form*. The fact that the milder diseases of this class prevail epidemically in the country, in the immediate vicinity of cities, simultaneously with the cholera in those cities, is explicable upon the same hypothesis. And this brings me to the question so frequently asked—is cholera contagious? I answer, that although I have seen a great many cases of cholera, in all stages of the disease and never had a symptom of the disease myself, yet I do believe the disease to be contagious. Less so, undoubtedly than smallpox, and many other diseases, yet certainly so to an appreciable degree; if by contagion is meant that the body of the diseased person is capable of eliminating a poison similar to that which produced the disease, thus adding to that already present in the atmosphere. The facts in support of this opinion are, I think, more numerous and conclusive than those opposed to it. Hence, when questioned upon this point I always advise, when practicable, an avoidance of the infected district, and particularly the houses in which persons have died of cholera.

I may mention, before closing this communication, that I have recently used the solution of camphor in chloroform in one case of hysteria, one of mania-â-potu, and one of severe cramp colic, and at each repetition have been more and more pleased with its effects.

Germantown, Tenn., July 10, 1849.

